# Editorial: Exploring lymphatic vasculature's role in cardiovascular and metabolic diseases

**DOI:** 10.3389/fcvm.2026.1805707

**Published:** 2026-03-04

**Authors:** Xiaolei Liu, Xiaofeng Yang, Michael V. Autieri

**Affiliations:** Lemole Center for Integrative Lymphatic and Vascular Research, Department of Cardiovascular Sciences, Lewis Katz School of Medicine at Temple University, Philadelphia, PA, United States

**Keywords:** atherosclerosis, endothelial cell, inflammation, lymphatic system, myocarditis

The lymphatic vasculature is present in almost every tissue in the body and runs in parallel to the blood vasculature but has distinct structural and functional roles. Whereas blood vasculature is a bidirectional circulatory system, the lymphatic system is a unidirectional circulatory structure that drains lymph fluid away from tissues to return it to the blood circulation. The lymphatic system is composed of two structurally and functionally unique vessel morphologies, each of which are comprised of lymphatic endothelial cells (LECs). The lymphatic system begins as blunt-ended initial lymphatics which contain button-like junctions allowing for passive uptake of lymph which then converge to form collecting lymphatics which maintain lymph within the system by relatively impermeable zipper junctions (1–3). To retain its unique structure and function, lymphatic vessels must maintain a rigorous and tightly regulated morphology of the junctional proteins located between lymphatic endothelial cells (3, 4). This structure-function relationship is important in disease states, as lymphatic vessels enlarge in inflamed tissue and the junctional morphology appears to reverse wherein initial lymphatics become zippered and collecting lymphatics become buttoned, contributing to overall reduced lymphatic functionality (5–8). This leads to poor lymphatic drainage of pro-inflammatory molecules and less leukocytes egression, exacerbating inflammation and preventing resolution (8). Despite its essential, central function, the lymphatic system remains understudied and its role in tissue homeostasis in general, and in cardiovascular diseases in particular is often overlooked and requires additional investigation and is the focus of this special issue.

In alignment with the concept that the lymphatic system is in a unique position to integrate multiple tissues and organs in health and disease, current understanding suggests that enhancement of lymphatic function has the potential to bridge systemic and organ-specific pathologies. In this way, increasing lymphatic function by increasing the lymphatic bed through lymphangiogenesis/lymphavasculogenesis, and/or lymphatic function by increasing permeability has therapeutic potential to combat cardiovascular and other diseases. Cardiovascular diseases (CVD) are the number one cause of death in the western world, and it is estimated that up to three-fourths of cardiovascular diseases are diseases of the vasculature. Despite recent advances in lipid-lowering therapy, atherosclerosis, a lipid-driven vascular inflammatory process remains the leading cause of death in high-income countries. The lymphatic system can participate in resolution and stabilization of existing plaque by facilitating reverse cholesterol transport; an high density lipoprotein (HDL)-mediated removal of cholesterol from arterial walls and its transport through the lymphatic system back to the liver for excretion in bile and feces. Atherosclerosis and other cardiovascular diseases are more prevalent in men than women, however, CVD risk rises sharply after menopause, and lymphedema studies suggest that females experience a more pronounced age-related decline in lymphatic function compared with males. An elegant study in this issue by Mesples et al*.* tests the hypothesis that hormonal changes throughout life affect lymphatic transport in individuals predisposed to CVD. This important study utilized ovariectomized females and males treated with 17β-estradiol (E2) and correlated lesion burden and improved lymphatic transport the expression of key lymphatic endothelial and muscle cell genes involved in vessel integrity and function. The exciting results of this study indicate that estrogens modulate lymphatic function and atherosclerosis differently according to sex and baseline hormonal status and suggest that lymphatic function may contribute to the interplay between hormonal changes and cardiovascular risk.

Cardiovascular-kidney metabolic syndrome (CKM) syndrome is a multi-factorial set of symptoms in which dysfunctional adipose tissue manifested by obesity and diabetes predisposes an individual to coronary artery and peripheral artery disease, heart, and renal failure. In this issue, the manuscript by Fowler et al. propose the hypothesis that enhancing lymphatic function may be a novel and effective approach to improve quality of life in patients with CKM syndrome by engaging multiple pathologies simultaneously throughout the body. They provide examples where administration of lymphangiogenic factors such as Vascular endothelial growth factor C (VEGFC) could influence the severity and outcomes of heart failure, atherosclerosis, chronic kidney disease, obesity and metabolic syndrome.

Myocarditis is an inflammatory disease of the heart muscle. It can be initiated by many diverse insults ranging from infectious agents and non-infectious causes such as autoimmune disease. In all cases, myocarditis is characterized by an immune reaction featuring leukocyte infiltration into the myocardium with associated cardiomyocyte fibrosis and necrosis. In this focused issue, Kurup et al. propose that in addition to the well-studied inflammatory mechanisms the pathophysiology of myocarditis may include mechanical stresses resulting from increased myocardial edema resulting from dysfunctional lymphatic clearance. In this scenario, the resulting accumulation of interstitial fluid fosters sustained inflammation, fibroblast activation, and extracellular matrix (ECM) remodeling that may stimulate fibrosis, stiffen the myocardium, and impair cardiac function. The chronic presence of myocardial edema can exacerbate electrical instability and promote conduction abnormalities that increase susceptibility to atrial fibrillation and ventricular tachycardia, which are common arrhythmic complications in patients with myocarditis. These authors propose that since lymphatic remodeling and subsequent edema is prevalent in patients with myocarditis, that in addition to anti-inflammatory and anti-infectious agents, therapeutically enhancing lymphatic function could limit the severity and progression of myocarditis. These concepts are strongly supported by parallel insights from studies of ischemic and non-ischemic heart disease, where cardiac lymphatic dysfunction has emerged as a key determinant of post-injury repair and adverse remodeling. Lymphatic-based therapies arise as an emerging frontier in the treatment of inflammatory and ischemic heart diseases.

Analogous to the Warburg effect in cancer cells, LECs generate nearly 70% of ATP via glycolysis instead of other metabolic processes such as oxidative phosphorylation, even in the presence of oxygen, a unique characteristic which differentiates LECs from many other cell types. The review by Simeroth et al. summarizes our current understanding of LEC metabolic processes. For example, several cellular metabolic pathways in lymphangiogenesis such as glycolysis, mitochondrial respiration, Ketone body oxidation (KBO), and fatty acid β-oxidation (FAO) are all rather unique in LECs and this has pathological implications for human diseases. It highlights how these LEC metabolic processes are implicated in various lymphatic pathologies such lymphedema, and how a better understanding of LEC metabolism can aid us in development of new treatments for human pathological conditions related to the lymphatic system.

Taken together, these studies highlight the central role of the lymphatic system in health and disease. They clearly indicate that modulation of lymphangiogenesis and lymphatic function can influence the development and severity of many cardiovascular diseases and that further investigation into the role of the lymphatic system in these diseases are essential for future development of novel therapeutics for cardiovascular diseases.
